# Supplementary figures and images for: Proteomic Profiling of Cardiomyocytes Revealed Potential Radioprotective Effects of Different Resveratrol Pretreatment Regimens
Source: Int J Mol Sci. 2025 Oct 21;26(20):10223. doi: 10.3390/ijms262010223 (PMC12563971; doi:10.3390/ijms262010223)

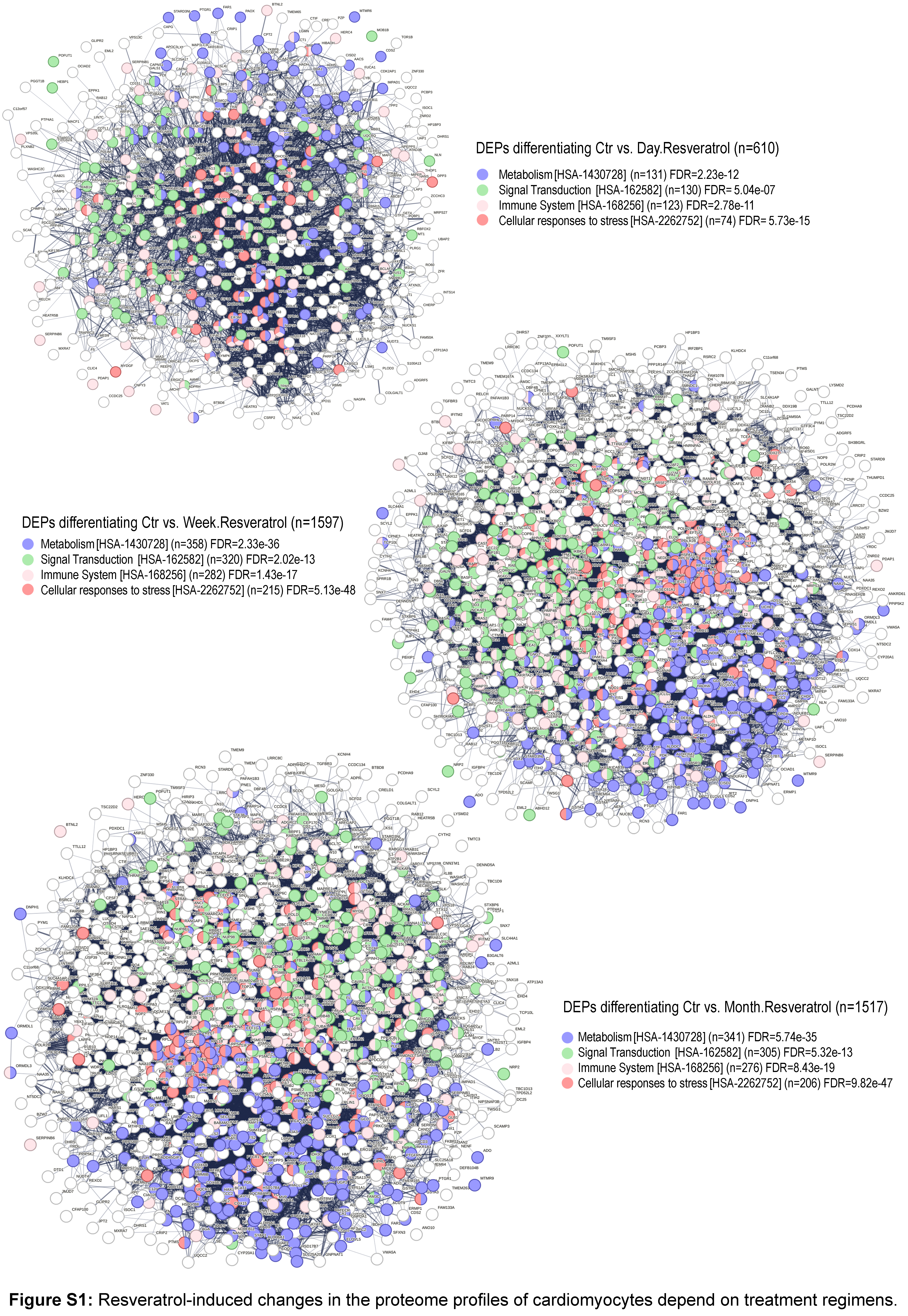

Supplement: Supplementary file 1 [file ijms-26-10223-s001.zip › Supplementary Figure S1'.png]
